# Evidence for a chemical arms race between cuckoo wasps of the genus *Hedychrum* and their distantly related host apoid wasps

**DOI:** 10.1186/s12862-022-02093-8

**Published:** 2022-11-28

**Authors:** Ruth Castillo, Mareike Wurdack, Thomas Pauli, Alexander Keller, Heike Feldhaar, Carlo Polidori, Oliver Niehuis, Thomas Schmitt

**Affiliations:** 1 Department of Animal Ecology and Tropical Biology, Biocentre, University of Würzburg, Am Hubland, 97074 Würzburg, Germany; 2 Department of Evolutionary Biology and Ecology, University of Freiburg, Hauptstraße 1, 79104 Freiburg, Germany; 3 Institute of Medical Bioinformatics and Systems Medicine, Medical Center, University of Freiburg, Breisacher Straße 153, 79110 Freiburg, Germany; 4 Cellular and Organismic Networks, Faculty of Biology, Ludwig-Maximilians-University München, 82152 Planegg-Martinsried, Germany; 5 Animal Population Ecology, Department of Animal Ecology I, Bayreuth Center of Ecology and Environmental Research (BayCEER), University of Bayreuth, 95440 Bayreuth, Germany; 6 Dipartimento di Scienze e Politiche Ambientali, Università degli Studi di Milano, via Celoria 26, 20133 Milan, Italy

**Keywords:** Chemical mimicry, Chrysididae, Cuticular hydrocarbons, Evolutionary arms race, Hymenoptera, Philanthidae

## Abstract

**Background:**

Brood parasites can exert strong selection pressure on their hosts. Many brood parasites escape their detection by mimicking sensory cues of their hosts. However, there is little evidence whether or not the hosts are able to escape the parasites’ mimicry by changing these cues. We addressed this question by analyzing cuticular hydrocarbon (CHC) profiles of *Cerceris* and *Philanthus* wasps and their brood parasites, cuckoo wasps mimicking the CHC profiles of their hosts. Some of these hosts use hydrocarbons to preserve their prey against fungal infestation and thus, they cannot significantly change their CHC composition in response to chemical mimicry by *Hedychrum* brood parasites.

**Results:**

We found that the CHC overlap between brood parasites and their hosts was lower in case of host wasps not preserving their prey than in case of prey-preserving host wasps, whose CHC evolution is constrained. Furthermore, the CHC profiles in non-preserving host wasps is more strongly diversified in females than in males, thus in the sex that is chemically mimicked by brood parasites.

**Conclusion:**

Our results provide evidence for a chemical arms race between those hosts that are liberated from stabilizing selection on their chemical template and their parasites.

**Supplementary Information:**

The online version contains supplementary material available at 10.1186/s12862-022-02093-8.

## Introduction

Coevolution between interacting species is considered one of the major forces generating biological diversity [1]. Coevolution plays an important role in the organization of communities, by shaping, for example, both symbiotic and parasitic interactions and reciprocal specialization among free-living taxa [2, 3], and in promoting the appearance of key innovations [4, 5]. Yet, demonstrating that a given trait has evolved in response to another trait from a different species and that the evolved trait caused counter-adaptations in return is not straightforward. It is particularly difficult to rule out effects of external selection pressures on a particular trait of interest [6].

Among antagonistic interactions, interspecific brood parasitism is a widespread strategy of kleptoparasites and parasitoids (subsequently collectively referred to as brood parasites). Brood parasites exploit their host’s provision for offspring (in case of kleptoparasites) or the immature stages of the host’s offspring itself (in case of parasitoids) to raise their own offspring [6–9]. One of the best studied examples of coevolution between brood parasites and their hosts is that between cuckoos (Aves: Cuculidae) and their hosts. Here, hosts of cuckoos evolved various strategies to compromise optical mimicry of host eggs by cuckoos, such as increased egg color polymorphism [6, 10]. For such antagonistic coevolutionary relationships, Van Valen’s Red Queen Hypothesis provides a theoretical framework that explains the evolutionary arms race between hosts and their parasites [11].

Olfactory communication plays a fundamental role for insects [12]. As virtually all insects are covered by cuticular hydrocarbons (CHCs), this diverse class of semiochemicals has gained particular importance for communicating important information, such as species identity, age, sex, reproductive status, and—in eusocial species—caste and colony membership [13]. It therefore comes as no surprise that some brood parasitic insects evolved the ability to chemically deceive their hosts in order to conceal their detrimental action.

Insect brood parasites are known to adopt one of at least three different strategies to successfully deceive their host’s olfactory recognition system [14]: (1) the brood parasite synthesizes a low total amount and/or a simplified blend of hydrocarbons on its cuticle and thus provides few recognition cues to the host [15–17]; this is called the *chemical insignificance* strategy. (2) The brood parasite physically adopts the host’s CHC profile by grooming; this strategy is referred to as *chemical camouflage* [18, 19]. (3) The brood parasite synthesizes de novo a CHC profile that is very similar to that of its host [14]; this strategy is known as *chemical mimicry*. These three strategies are not mutually exclusive, and some brood parasites apply them simultaneously. For example, the butterfly *Phengaris (Maculinea) rebeli*, whose larvae develop in ant nests, applies both a chemical mimicry and a chemical camouflage strategy to deceive its host [20].

Hosts can counteract the chemical deception strategies of their brood parasites by evolving an improved ability to recognize and to discriminate chemical cues and/or by expressing chemical phenotypes that differ from those of their brood parasites (*e.g.*, via negative frequency-dependent selection; [21, 22]. However, the evolutionary response by hosts to chemical mimicry by their brood parasites has received comparatively little attention so far.

Despite the importance of chemical mimicry for insect brood parasites, very few studies have provided evidence for this strategy to result in a coevolutionary arms race between brood parasites and their hosts. Examples include slave-making ants [23–25], bumble bees [26], vespid wasps [27], and cuckoo wasps and their hosts [21, 28]. In most of the studied cases, the brood parasitic species apply a chemical camouflage strategy (*i.e.*, ants, bumble bees, vespid wasps), often in association with the insignificance strategy. Furthermore, most of these cases involve host-brood parasite species pairs that are phylogenetically closely related, a phenomenon known as Emery’s rule [29]. Clear evidence for chemical mimicry is currently only known from host-brood parasite species pairs that are phylogenetically distantly related. Here, the brood parasites have been forced to evolve a chemical profile that matches that of their hosts [20, 21].

To shed light on chemical adaptations in host-brood parasite species pairs, we investigated coevolutionary patterns in CHC profiles across species, using cuckoo wasps of the genus *Hedychrum* (Hymenoptera: Chrysididae: Elampini) and their distantly related hosts, apoid wasps of the genera *Cerceris* and *Philanthus* (Hymenoptera: Philanthidae) [30–33], as models. Females of *Cerceris* and *Philanthus* species dig brood cells in the ground and provision their larvae with paralyzed prey, which consists either of beetles (Coleoptera-hunting wasps: COLw) or of stinging wasps and bees (Hymenoptera-hunting wasps: HYMw).

What makes the above mentioned hosts of *Hedychrum* cuckoo wasps particularly interesting is the fact that females of HYMw (but not of COLw) are known to embalm their prey with a secretion from their postpharyngeal gland to delay/prevent fungal infestation of the prey [34–37]. These secretions consist primarily of unsaturated long-chain hydrocarbons (*e.g.*, alkenes), which form a hydrophobic oily layer on the prey that prevents water condensation and impairs mold development on the prey [38, 39]. Moreover, the hydrocarbon composition of the postpharyngeal gland content of female HYMw strongly matches the CHC composition of the wasps’ own cuticle [40], similar to what was previously observed in ants [41]. As the CHC composition of the postpharyngeal gland content is highly similar among species, the specific composition likely represents an adaptation to preserve prey and the composition is thus likely under stabilizing selection [37]. In contrast, females of COLw do not embalm their prey with a secretion of CHCs, because their prey is less susceptible to fungal growth [37]. Females of HYMw consequently cannot alter their CHC profile without losing the ability to preserve their prey. In contrast, females of COLw do not seem to be constrained by stabilizing selection on their CHC profile and thus have the ability to shift their CHC profiles to avoid chemical mimicry by their brood parasites [37].

In this study, we report results from testing three hypotheses: (1) CHC profile mimicry is less precisely accomplished by cuckoo wasps that are brood parasites of COLw than by those that are brood parasites of HYMw, because COLw have more abilities to alter their CHC profiles in response to chemical mimicry than HYMw have. (2) Only female cuckoo wasps apply a chemical mimicry strategy, because only females benefit from not leaving detectable traces of their intrusion in the host nest. (3) Possible chemical counter-adaptations by the hosts should primarily affect females, whose nests are intruded by cuckoo wasps. This is because only female hosts are under selective parasite pressure to evolve counter-adaptations to chemical mimicry by cuckoo wasps. To be better able to interpret possible adaptations and counteradaptations in the CHC profiles of hosts and parasites, we considered the phylogenetic history of the investigated species by inferring the phylogeny for the genus *Hedychrum* and considering a published phylogenetic tree of the hosts.

## Results

### Molecular phylogeny and evolutionary relationships

**Fig. 1:**
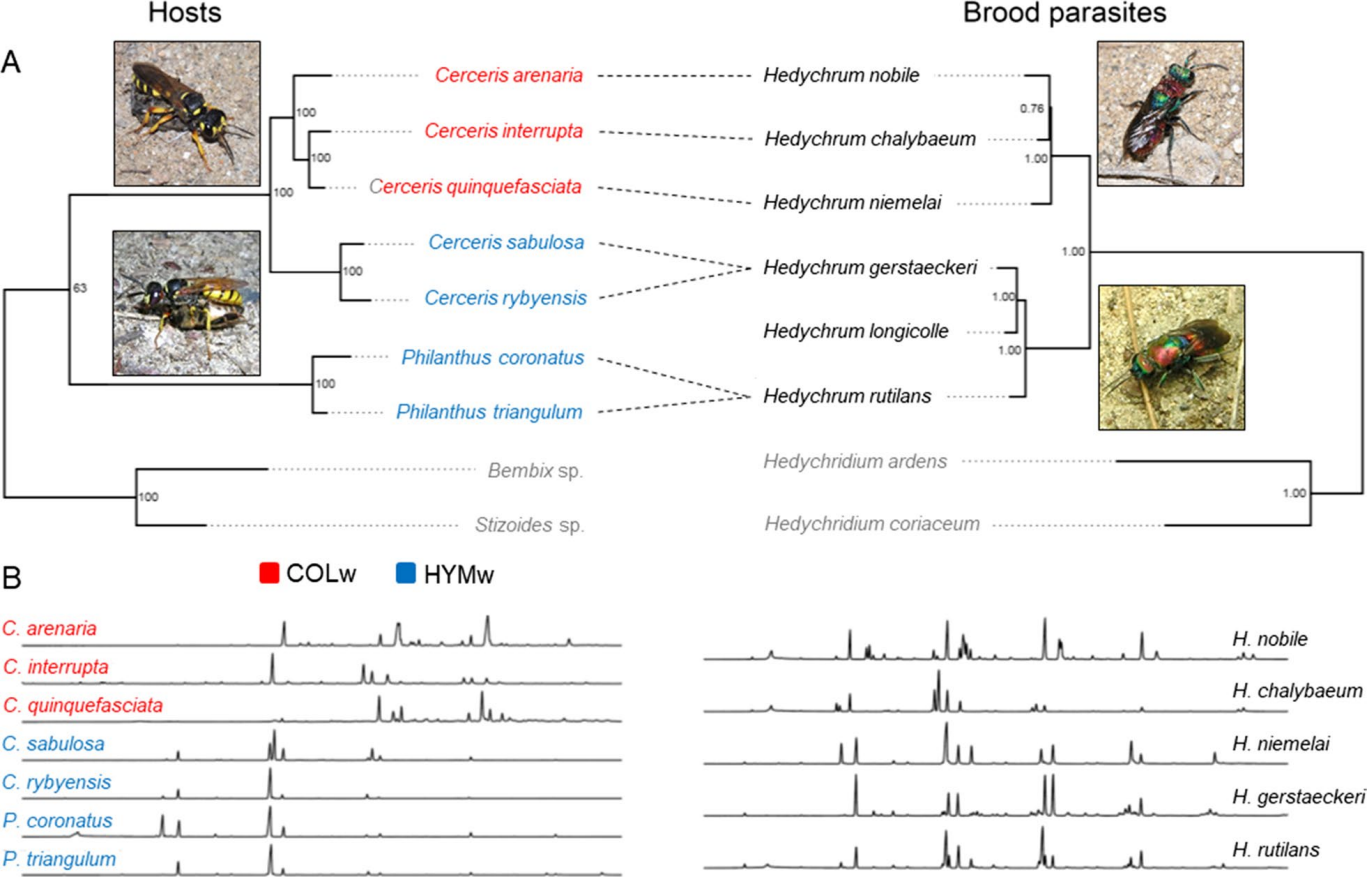
**A** Phylogenetic relationships of the analyzed hosts (left, adapted from Wurdack et al*.*, 2017) and their brood parasites (right). Pictures show a female *Cerceris arenaria* with weevil prey (up left), a female *Philanthus triangulum* with honeybee prey (down left), a female *Hedychrum nobile* at *C. arenaria* nesting site (up right) and a female *Hedychrum rutilans* at *P. triangulum* nesting site (down right). **B** Representative chromatograms of female individuals of the hosts (left) and brood parasites (right). The phylogenetic trees were inferred using the maximum likelihood optimality criterion. Bootstrap support values are given above each node. Host-brood parasite relationships are indicated, except for outgroup species (in grey) (*Stizoides* sp. and *Bembix* sp., and *Hedychridium* spp.) and *Hedychrum longicolle*

Our inferred cuckoo wasp phylogeny shows that the species parasitizing COLw (*Hedychrum chalybaeum*, *Hedychrum niemelai*, and *Hedychrum nobile*) and the species parasitizing HYMw (*Hedychrum gerstaeckeri*, *Hedychrum longicolle*, and *Hedychrum rutilans*) each form a natural group. In contrast, the host species that hunt Hymenoptera are paraphyletic with respect to the host species that hunt Coleoptera [37] (Fig. 1).

### Cuticular hydrocarbon profile composition

**Fig. 2:**
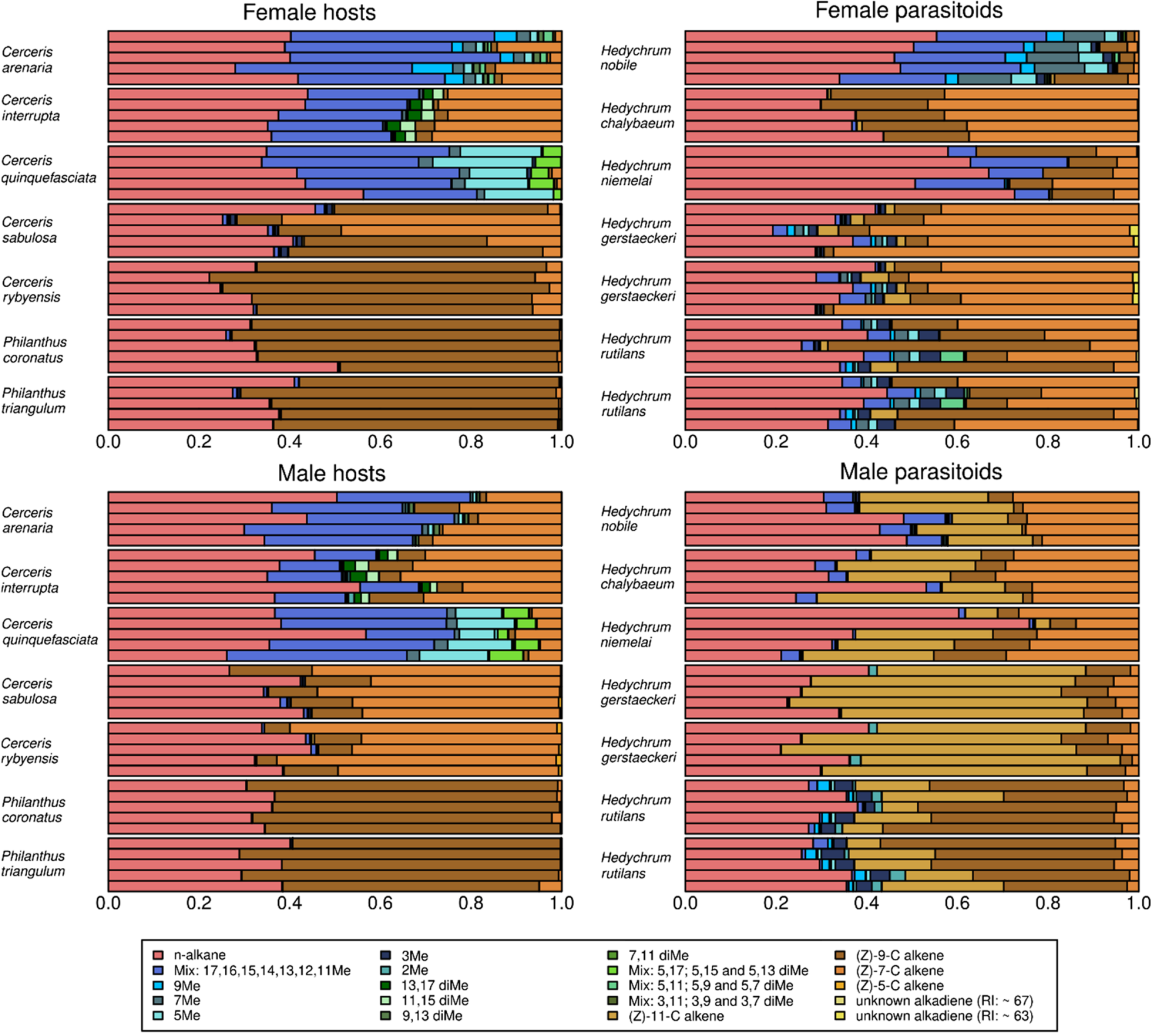
Composition of CHC profiles sorted according to the most common functional groups in the analyzed species. Left columns refer to hosts and right columns refer to brood parasites; top graphs refer to females, bottom graphs to males. Five randomly selected samples of each species and sex illustrate within-group CHC variability. Color hues indicate different CHC classes, also sorted from right to left: pink indicates pure n-alkane (C21–C33), blues are used to indicate monomethyl-branched alkanes, greens indicate dimethyl-branched alkanes, orange-brown colors are used to indicate alkenes and yellow hues indicate alkadienes

In total, we identified 112 different CHCs across the two sexes of the twelve species (hosts and brood parasites) (N = 277; Additional file 2: Tables S4,S5). Among the 112 CHCs, twelve are linear alkanes, 30 are alkenes, nine are alkadienes, 39 are monomethyl- and 22 are dimethyl-branched alkanes. Linear alkanes constitute 30–45% of the total CHC amount in each species (except in females of *H. niemelai*, whose alkanes make up 64% of the total CHC amount). Unsaturated compounds comprise up to 65% of a given CHC profile of Hymenoptera-hunting wasps/hosts (HYMw), whereas the proportion of methyl-branched alkanes in HYMw is less than 3% of the total CHCs (Table 1). We found no dimethyl-branched alkanes in any HYMw. In contrast, methyl-branched alkanes range from 20% (males of *Cerceris interrupta*) to more than 60% of the CHC profile of COLw (Fig. 2).

**Table 1:** Number of CHCs and relative proportions of hydrocarbon substance classes of studied species and sex

Species	Sex	Wasp	Sample size	Number of CHC	Methyl-branched Alkanes	Unsaturated CHC	n-Alkanes
*Cerceris arenaria*	Female	COLw	8	36 ± 1	52.1 ± 0.06	9.4 ± 0.07	38.5 ± 0.04
*Cerceris interrupta*	Female	COLw	8	34 ± 1	30.9 ± 0.03	29.7 ± 0.04	39.4 ± 0.04
*Cerceris quinquefasciata*	Female	COLw	14	24 ± 4	60.8 ± 0.09	0.3 ± 0.0	38.9 ± 0.09
*Cerceris rybyensis*	Female	HYMw	14	21 ± 2	0.1 ± 0.0	65.2 ± 0.11	34.7 ± 0.11
*Cerceris sabulosa*	Female	HYMw	14	27 ± 3	2.5 ± 0.01	60.6 ± 0.07	36.9 ± 0.07
*Hedychrum chalybaeum*	Female	brood parasite	9	24 ± 0	0.2 ± 0.0	63.2 ± 0.05	36.6 ± 0.05
*Hedychrum gerstaeckeri*	Female	brood parasite	14	36 ± 5	5.3 ± 0.04	64.7 ± 0.05	30.0 ± 0.05
*Hedychrum niemelai*	Female	brood parasite	11	20 ± 2	13.8 ± 0.09	22.2 ± 0.09	64.0 ± 0.1
*Hedychrum nobile*	Female	brood parasite	13	38 ± 2	47.8 ± 0.05	9.4 ± 0.06	42.8 ± 0.08
*Hedychrum rutilans*	Female	brood parasite	14	42 ± 3	15.0 ± 0.08	43.7 ± 0.14	41.3 ± 0.1
*Philanthus coronatus*	Female	HYMw	5	24 ± 2	0.2 ± 0.0	65.4 ± 0.09	34.4 ± 0.09
*Philanthus triangulum*	Female	HYMw	9	26 ± 2	0.5 ± 0.0	64.9 ± 0.04	34.6 ± 0.04
*Cerceris arenaria*	Male	COLw	11	31 ± 1	32.8 ± 0.06	26.3 ± 0.06	40.9 ± 0.1
*Cerceris interrupta*	Male	COLw	6	38 ± 1	19.5 ± 0.04	37.8 ± 0.06	42.7 ± 0.08
*Cerceris quinquefasciata*	Male	COLw	11	36 ± 3	54.5 ± 0.1	7.7 ± 0.05	37.8 ± 0.09
*Cerceris rybyensis*	Male	HYMw	14	26 ± 3	1.1 ± 0.01	62.1 ± 0.04	36.9 ± 0.04
*Cerceris sabulosa*	Male	HYMw	14	23 ± 5	1.2 ± 0.01	63.9 ± 0.06	34.9 ± 0.06
*Hedychrum chalybaeum*	Male	brood parasite	10	31 ± 1	4.3 ± 0.01	62.4 ± 0.08	33.3 ± 0.08
*Hedychrum gerstaeckeri*	Male	brood parasite	14	22 ± 2	0.6 ± 0.01	67.4 ± 0.1	32.0 ± 0.1
*Hedychrum niemelai*	Male	brood parasite	10	24 ± 1	1.8 ± 0.01	53.9 ± 0.16	44.2 ± 0.16
*Hedychrum nobile*	Male	brood parasite	14	34 ± 1	8.0 ± 0.02	51.3 ± 0.1	40.7 ± 0.1
*Hedychrum rutilans*	Male	brood parasite	14	34 ± 3	8.5 ± 0.03	58.3 ± 0.06	33.2 ± 0.07
*Philanthus coronatus*	Male	HYMw	14	20 ± 1	0.0	63.6 ± 0.05	36.4 ± 0.05
*Philanthus triangulum*	Male	HYMw	12	18 ± 1	0.0	61.5 ± 0.05	38.5 ± 0.05

Average values ± standard deviations are shown

The CHC profiles of female HYMw are very similar to each other across species and are predominantly composed of alkenes (60–65%) and linear alkanes (34–37%). Males of HYMw species are similar in their CHC substance class composition to their female conspecifics (Fig. 2), but they systematically differ from conspecific females by a high relative abundance of alkenes that exhibit their double bonds at positions different from those of alkenes of the females. In contrast, females and males of COLw are more dissimilar in their composition of alkenes (0–30% and 8–38% in females and males, respectively) and methyl-branched alkanes (31–61% and 20–55% in females and males, respectively). Females of COLw possess a larger proportion of methyl-branched alkanes in their CHC profiles than their conspecific males (50% vs. 38%, t(54) = 3.03, *p* = 0.049; Table 3; Additional file 1: Fig. S1c). In general, both the CHC profiles of female and male HYMw possess less diverse CHC profiles (with an average number of 24.7 ± 3.49 and 21.9 ± 4.46 CHC compounds in the profiles of females and males, respectively) than CHC profiles of female and male COLw (average number of 30.0 ± 6.17 and 34.5 ± 3.65 CHC compounds in the profiles of females and males, respectively). The lower number of compounds in HYMw than in COLw is due to the very low number of methyl-branched compounds that HYMw express (on average fewer than six compounds, Additional file 2: Tables S4,S5). Among COLw, only *Cerceris arenaria* females possess more compounds in their CHC profiles than males (35.9 ± 1.13 and 31 ± 1.26 in females and males, respectively; t(17) = 8.8427, *p* < 0.001). In the two other COLw species (*i.e.*, *Cerceris interrupta* and *Cerceris quinquefasciata*), males possess a larger number of CHC compounds in their CHC profiles than their conspecific females (Table 3; Additional file 1: Fig. S1a,b).

The profiles of cuckoo wasps contain all CHC substance classes found in their hosts. Species that parasitize HYMw feature larger proportions of alkenes compared to species parasitizing COLw (t(108) = − 4.99, *p* < 0.001). Species that parasitize COLw tend to show larger proportions of methyl-branched alkanes than HYMw except for *H. chalybaeum* (t(88) = − 2.88, *p* = 0.059). The latter species exhibits larger proportions of alkenes (> 60% of its CHC profile) similar to *Hedychrum* species parasitizing HYMw. Female brood parasites of COLw produce high relative amounts (14–48%) of methyl-branched alkanes except for *H. chalybaeum* (< 0.5%), while conspecific males produce high relative amounts (51–62%) of alkenes, similar in their proportion to the proportion of alkenes in the profiles of female and male brood parasites of HYMw (Fig. 2 and Table 1). Female and male brood parasites of HYMw differ from each other in the type of synthesized alkenes: females synthesize primarily alkenes with double bonds at position 7 and position 9, while conspecific males synthesize mainly alkenes with double bonds at position 9 and position 11.

**Fig. 3:**
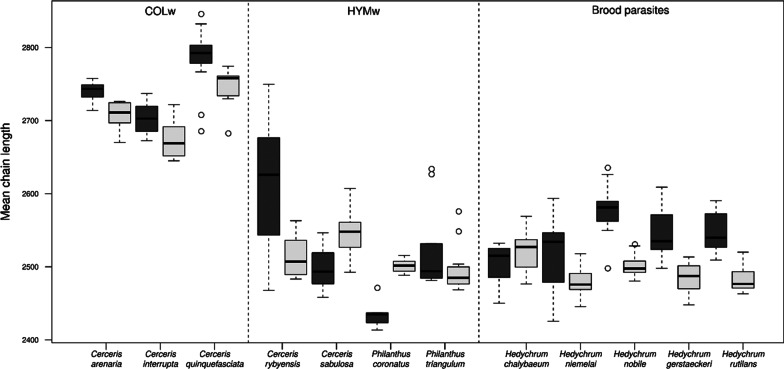
Mean chain length (expressed in RI) of the different species in this study separated by sex: females (dark gray) and males (light gray)

The average CHC chain length in COLw in both sexes is approximately two carbon atoms longer than that in HYMw and in all investigated brood parasites (Fig. 3). Both females and males of COLw species synthesize significantly less relative amounts of hydrocarbons with chain lengths of fewer than 24 carbon atoms than HYMw (t(97) = − 16.7, *p* < 0.001). In general, females of hosts and parasites synthesize CHCs with a longer mean chain length than their males (t(259) = 3.58, *p* = 0.007). When investigating different guilds (COLw, HYMw, and brood parasites) separately, this pattern was found when comparing males and females of COLw hosts (t(54) = 3.23, *p* = 0.029) and when comparing males and females of brood parasites (t(96) = 7.99, *p* < 0.001). This pattern was not found when comparing males and females of HYMw (t(50) = 1.47, *p* = 0.7).

### Chemical profile overlap between brood parasites and hosts

**Fig. 4:**
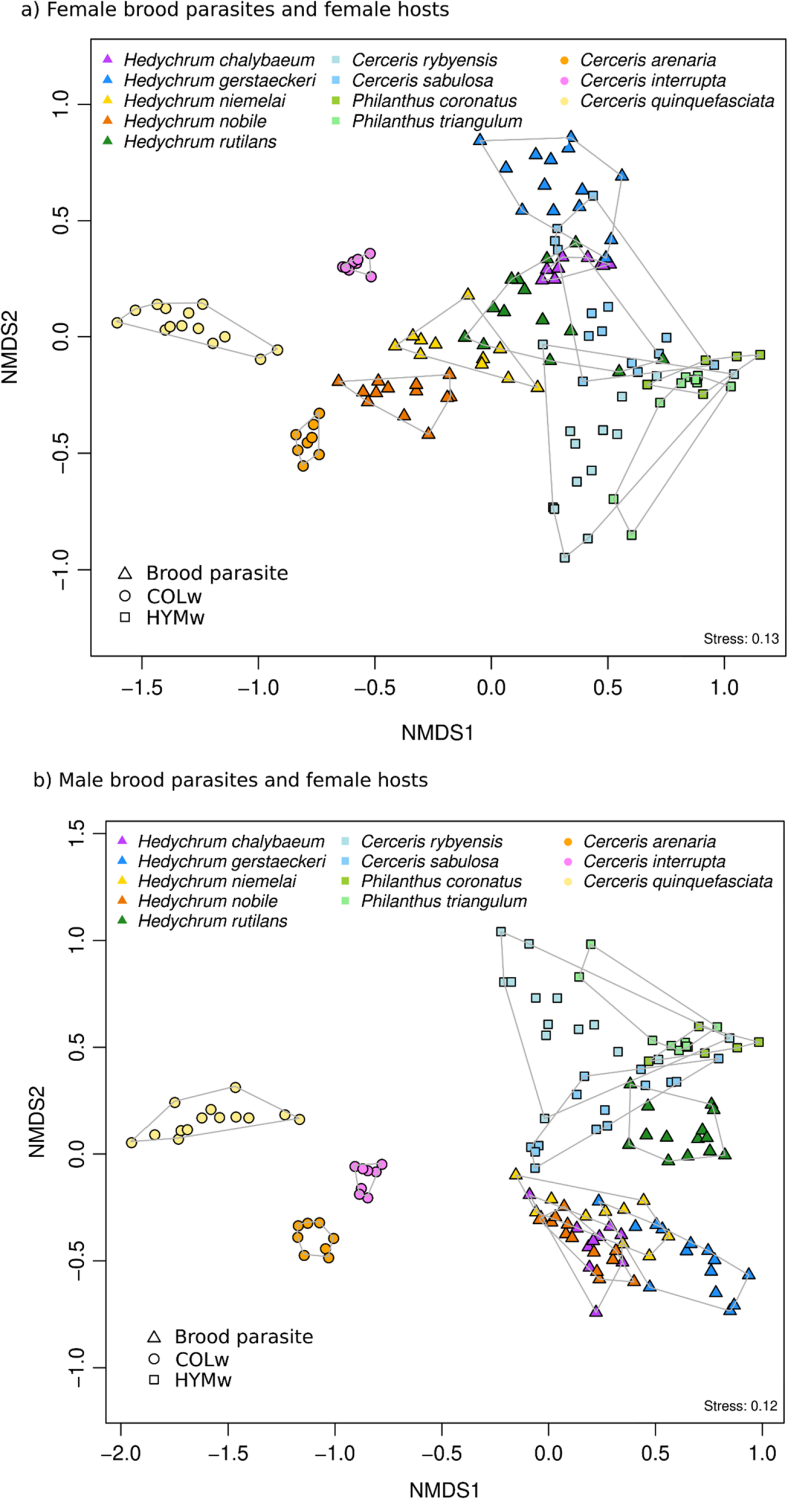
Separation of CHC profiles of the studied species by Non-metric Multidimensional Scaling. Coleoptera hunters (COLw) are indicated by circles, Hymenoptera hunters (HYMw) by squares and brood parasites by triangles. Similar hue colors are used to indicate host*-*brood parasites species pairs: orange (*Cerceris arenaria* / *Hedychrum nobile*), purple (*C. interrupta* / *H. chalybaeum*), yellow (*C. quinquefasciata* / *H. niemelai*), green (*Philanthus coronatus* and *P. triangulum* / *H. rutilans*), blue (*C. rybyensis* and *C. sabulosa* / *H. gerstaeckeri*). **a** Female brood parasites and their female hosts and **b** male brood parasites and their female hosts

NMDS revealed a clear separation of COLw and HYMw based on CHC profile data, with the two host types forming distinct groups with little overlap of their chemical space (Fig. 4a, ANOSIM R: 0.943, mean rank distance within groups: 685, mean rank distance between groups: 1890, *p* = 0.0029, 9999 permutations). This separation mainly results from differences in the relative amounts of methyl-branched compounds and of unsaturated compounds in both types of hosts (*i.e.*, COLw and HYMw). *Hedychrum nobile* and—to a lesser extent—*H. niemelai* produce large amounts of methyl-branched compounds and are therefore separated from the other cuckoo wasps by occupying a chemical space that falls between that of their hosts and that of HYMw. In contrast, all HYMw and their brood parasites occupy a comparatively small range of chemical space and exhibit overlapping chemical signatures (*i.e.*, they are quite similar to each other, Fig. 4a). An R value close or identical to 1 indicates no overlap between groups. Indeed, the R statistic of ANOSIM is larger than 0.99 in all COLw-brood parasites comparisons, whereas it ranges between 0.66 and 0.93 when comparing HYMw and their brood parasites. A different pattern was observed when the CHC profiles of male brood parasites were plotted with those of their female hosts: males of cuckoo wasps that parasitize COLw species are chemically similar among each other and separated from their COLw hosts (Fig. 4b, ANOSIM among male brood parasites of COLw hosts, R: 0.4873, *p* = 0.0029; ANOSIM between male brood parasites of COLw and their female COLw hosts, R: 1, *p* = 0.0029), whereas female brood parasites of COLw are very distinct from each other (ANOSIM R: 0.9686, *p* = 0.0029). With the exception of *H. rutilans*, male cuckoo wasps overlap more in their chemical profiles among each other than their females do (ANOSIM between female brood parasites of COLw and HYMw, R: 0.4524, *p* = 0.0029; ANOSIM between male brood parasites of COLw and HYMw, R: 0.7184, *p* = 0.0029). The overlap between female COLw and their female brood parasites is larger than that between female COLw and male parasites (ANOSIM between female COLw and their female brood parasites, R: 0.5873, *p* = 0.0029; ANOSIM between female COLw hosts and their male brood parasites, R: 0.9349, *p* = 0.0029). However, when analyzing HYMw, the chemical space overlap between female brood parasites of HYMw and their female hosts is similar than that between male brood parasites of HYMw and female hosts (ANOSIM between female HYMw and their female brood parasites, R: 0.4297, *p* = 0.0029; ANOSIM between female HYMw hosts and their male brood parasites, R: 0.4651, *p* = 0.0029).

**Fig. 5:**
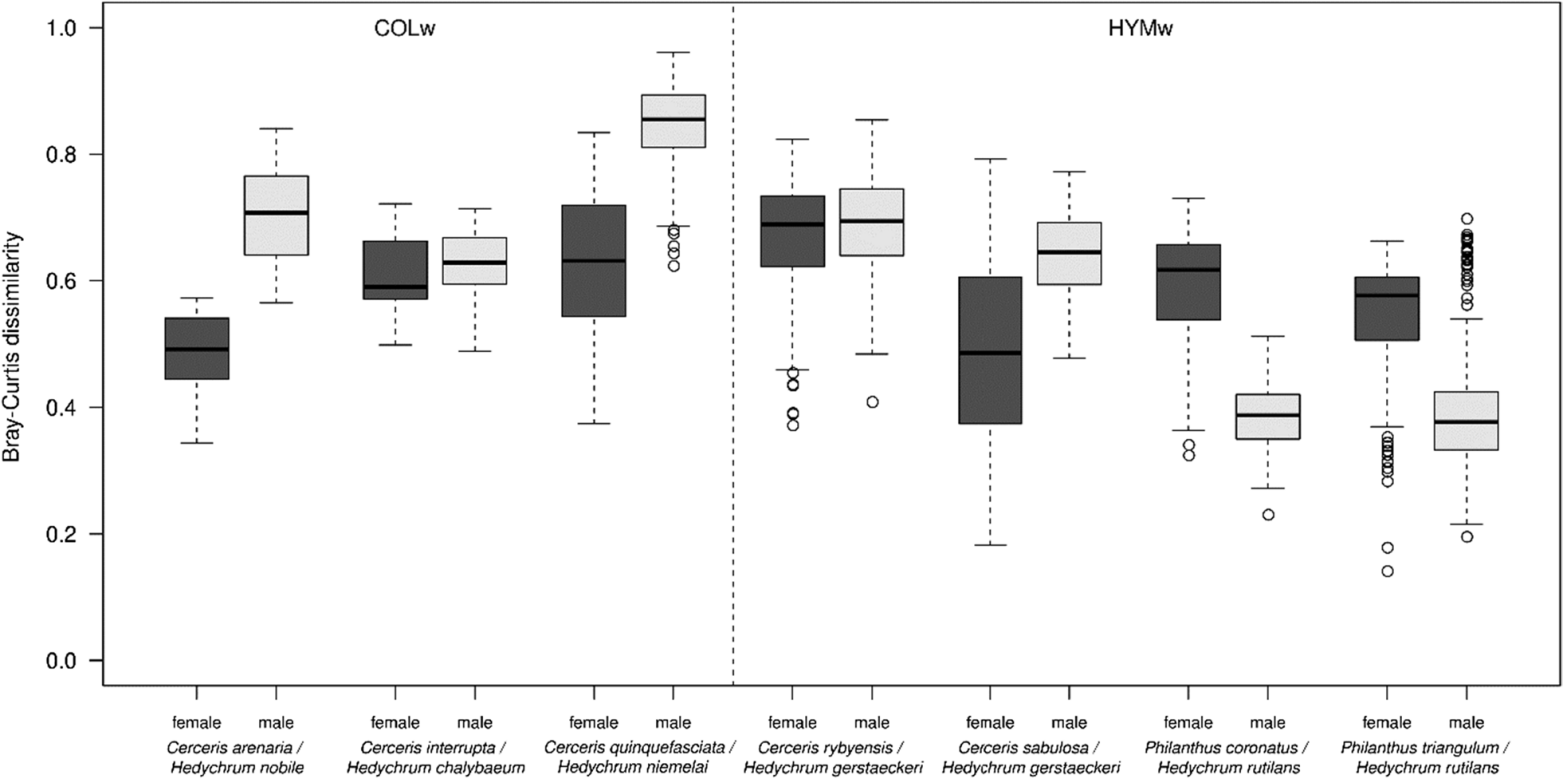
Host-brood parasite Bray–Curtis dissimilarities for COLw and HYMw. Dark gray boxes indicate female host-female brood parasite distances, light gray boxes female host-male brood parasite distances

Female host/female brood parasite chemical distances were almost always smaller than female host/male brood parasite chemical distances (Bray–Curtis dissimilarity index calculated across all compounds present). Exceptions were comparisons between the two *Philanthus* species and *H. rutilans* (Fig. 5). We did not find a difference between the chemical distances of female HYMw hosts to their female brood parasites and between the chemical distances of female COLw hosts to their female parasites (t(820) = − 1.36, *p* = 0.77). Chemical distances of COLw female hosts to their female brood parasites were smaller than the chemical distances to their male brood parasites (0.58 vs. 0.68, t(643) = − 10, *p* < 0.001). However, chemical distances of HYMw female hosts to their female brood parasites were similar to those of their male brood parasites (0.57 vs. 0.58; t(1,191) = 1.84, *p* = 0.5) (Fig. 5).

### Intra- and interspecific CHC variability of HYMw and COLw hosts

**Fig. 6:**
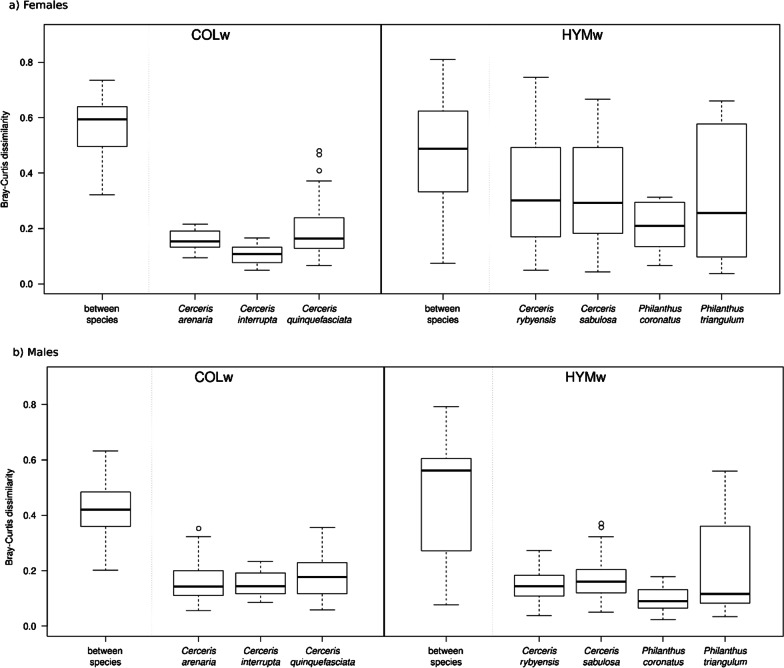
Intra- and interspecific variability of cuticular hydrocarbon profiles in **a** female and **b** male individuals of all host species. Bray–Curtis dissimilarities were calculated between all individuals of species hunting the same type of prey (“between species”) and between individuals of the same species (species name indicated). COLw: Coleoptera-hunting wasps; HYMw: Hymenoptera-hunting wasps

We observed a pattern indicating differences in inter- and intraspecific variability of CHC profiles in the NMDS plot (Fig. 4a). To further assess this pattern, we calculated Bray–Curtis dissimilarities between and within species. Interspecific differences were significantly larger between females of COLw hosts than between females of HYMw hosts (average 0.57 ± 0.09 vs. 0.47 ± 0.17; Bray–Curtis distance; t(897) = − 11.39, *p* < 0.001). In contrast, intraspecific variability was smaller between females of COLw hosts than between females of HYMw hosts (on average, Bray Curtis distances between samples within COLw species were 0.17 ± 0.08; those within HYMw species were 0.32 ± 0.19; t(334) = 10.83, *p* < 0.001). Hence, in contrast to females of COLw host species, females of HYMw host species show a larger intraspecific profile variance and thus a much larger dissimilarity between individuals belonging to the same species. This trend was not observed in conspecific males (Fig. 6). Males of COLw and HYMw show a smaller intraspecific variability relative to conspecific females. To test whether these results are not an artifact of sample size differences among species, we repeated the analyses using only five samples per species (the maximum number of samples consistently available across all analyzed species) and obtained comparable results (see Additional file 1: Fig. S2). In addition, we analyzed randomly picked CHC profiles of two females of a HYMw host species and of two females of COLw host species, with each pair of samples coming from the same population (same species and same locality of collection). We found that intraspecific differences in HYMw females are significantly larger than in COLw females [t(1324) = 22.2, *p* < 0.001]. By contrast, when repeating the analysis using the CHC profiles of two HYMw males and of two COLw males, no such differences were observed (see Additional file 1: Fig. S3).

## Discussion

The results of our study confirm the existence of clear CHC profile composition differences between COLw and HYMw, first suggested by Wurdack and colleagues [37]. HYMw species exhibit profiles that consist primarily of unsaturated compounds and that are more similar among each other than those of COLw species. In contrast, COLw species possess CHC profiles with a large number of mono- and dimethyl-branched alkanes, and these compounds also represent a substantial fraction of the total CHC amount. The compositions of the CHC profiles of all investigated species are species- and sex-specific and heritable due to their genetic basis of their biosynthetic pathways [13, 42]. Although we know that CHC profiles are plastic in some species, we can rule out plasticity as an explanation for the CHC differences to a large extent as we collected all individuals from the same area with very similar environmental conditions.

### CHC diversification in COLw has led to less chemical overlap between COLw and their brood parasites

Our first hypothesis stated that brood parasites of COLw species should chemically mimic their hosts’ CHC profile less precisely than brood parasites of HYMw. This is because COLw can change their CHC profile to a large extent since it is less constrained due to the abandonment of prey embalming, whereas HYMw seemingly cannot alter their CHC composition significantly at all, the latter thereby representing an easy target to chemically mimic. Brood parasites of HYMw should thus show a strong overlap in their CHC profile composition with that of their host, since HYMw are constrained to produce a relatively fixed and alkene-enriched CHC profile to cope with prey embalming. The abandonment of prey embalming behavior, which according to [37] likely evolved later in COLw, may have liberated COLw from producing alkene-enriched CHC profiles by relaxation from stabilizing selection.

We showed that COLw hosts differ in a number of features from HYMw hosts, all of which could be attributed to adaptations to escape mimicry by cuckoo wasps: (a) the biosynthesis of a larger number and higher amounts of methyl-branched CHCs, (b) the production of CHC compounds of higher chain length and reduction of shorter chain-length compounds, and (c) smaller intraspecific variation of CHC profiles in comparison to HYMw hosts (Fig. 6a). Moreover, the high between species dissimilarity observed in COLw could have arisen to avoid overlapping CHC profiles with their parasites but higher interspecific differences between COLw (Fig. 6a) to avoid overlapping CHC profiles with their parasites. As expected, there is a larger CHC profile overlap between HYMw and their female brood parasites but there is no overlap of CHC profiles between COLw and their female brood parasites. In contrast to our expectation, however, Bray–Curtis dissimilarities between HYMw and their brood parasites were not smaller than those between COLw female hosts and their brood parasites.

Female brood parasites of HYMw produce similar amounts of the same compound classes as their hosts [21]. However, differences in the double bond positions of alkenes in hosts and brood parasites contribute to larger chemical distances between the CHC profiles of female HYMw and their female brood parasites and can be interpreted as a strategy applied by HYMw to escape chemical mimicry. In addition, CHC profiles of female brood parasites of HYMw (*i.e.*, *H. gerstaeckeri* and *H. rutilans*) contain low amounts (~ 5%) of compounds (*e.g.*, methyl-branched compounds) that are absent in the CHC profile of their hosts and which contributes to the relatively large chemical distances observed between female HYMw and their female brood parasites. However, whether CHC compounds occurring at low abundances on the cuticle of HYMw brood parasites can be recognized by their hosts is unknown, especially when considering that these cues may interfere with scents of the environment (*e.g*., nest material, prey items provided).

Interestingly, although there is no overlap in the chemical space of the CHC profiles between COLw and their associated brood parasites, female brood parasites of COLw hosts (with the exception of *H. chalybaeum*) often produce the same methyl-branched compounds as their hosts (or at least methyl-branched compounds with the same branching position). The similarities in methyl-branched compounds between hosts and brood parasites are mainly responsible for the relatively short chemical distances between COLw hosts and their cuckoo wasps. A diversification of the CHC profiles with methyl-branched compounds may have resulted from selection on the hosts. In fact, some studies suggest that CHC profiles with more methyl-branched alkanes prevail in highly parasitized populations of other Hymenoptera: for example, parasitized ant colonies of *Formica fusca* show a higher diversity of dimethyl-branched alkanes than non-parasitized ones, and this increase in compound diversity correlates with increased recognition abilities in the host populations [43]. Likewise, the proportion of methyl-branched hydrocarbons in CHC profiles of the paper wasp *Polistes biglumis* is larger in populations highly parasitized by the social parasite *Polistes atrimandibularis* [44].

Apart from the diversification of the CHC profile in COLw, we found that the mean chain length of CHCs in COLw profiles is significantly larger than in the profiles of HYMw and those of all investigated brood parasites. Thus, COLw and their brood parasites differ in mean chain length from each other, while HYMw and their parasites do not. Several studies showed that shifts in chain-length in CHC profiles can be due to changes in climatic conditions (*e.g.*, [45–47]). However, since both types of hosts inhabit similar habitats (warm/dry conditions) and even co-occur in mixed nest aggregations—at least in the regions where we studied the species—the differences in the CHC chain lengths between the two groups of hosts are unlikely the result of differential adaptation to climatic conditions. It appears more likely that HYMw species require alkenes of a specific chain length, because this chain length allows maintaining a semifluid texture in the secretions that are spread on their prey against fungal infestation [39]. Thus, it is possible that the elimination of short-chain compounds in COLw may represent an additional escape strategy from chemical mimicry, as this increases the qualitative differences between the CHC profiles of hosts and parasites. A shift of the mean chain length could be beneficial if it allows the hosts to better discriminate between otherwise similar CHC profiles and thus allows them to detect differences between the CHC profiles of a potential brood parasite and their own. Moreover, an increase of the mean chain length of CHC would likely not require the evolution of new enzymatic pathways [48].

As mentioned above, the CHC profile similarity between HYMw and their brood parasites was lower than expected. We hypothesize that HYMw host counteract chemical mimicry of their brood parasites by applying an alternative evasion strategy. Chemically, HYMw are restrained to maintain the same proportion of alkenes in their CHCs because of the advantages alkenes confer to prey preservation. However, the CHC profiles of females of HYMw have a larger within-species variability than those of the females of COLw. In case of HYMw, negative frequency-dependent selection may favor the existence of rare host chemotypes. Highly variable CHC profiles could result in some individuals in a population exhibiting chemotypes that are likely not being well mimicked by a brood parasite. A similar phenomenon (*i.e*., increasing within clutch variation) has been observed in hosts of avian cuckoos [10]: females of some host species of cuckoos have evolved the ability to lay eggs with different color hues. It has been suggested that hosts of cuckoo that are in an evolutionary arms race with their brood parasites should increase inter-clutch variation (*i.e*., differences among eggs within a population, eggs laid by different females), but at the same time should keep intra-clutch color variation low [49] in order to make mimicry more difficult for the brood parasites. Similar adaptations for increasing within species variation in chemical signals have been observed in insects. For example, within population variation in the relative proportion of different CHCs is higher in highly parasitized populations of *Polistes biglumis* than in non-parasitized populations of this species, probably because of negative frequency-dependent selection of rare phenotypes [44]. Similarly, when the CHCs of *Temnothorax longispinosus* ants are compared within and between colonies in populations with and without the slave-making ant *Protomognathus americanus*, the CHC profiles of the host ant species are more variable in parasitized populations [22]. We have shown that two randomly chosen CHC profiles of a HYMw female host coming from the same population are more dissimilar to each other than two randomly chosen CHCs of a COLw host (Additional file 1: Fig. S3). CHC variation in HYMw females is largely quantitative. For example, in two out of nine female individuals of *Philanthus triangulum* in our analysis, the main compound being produced was (Z)-9-C27:1, whereas in the remaining individuals, (Z)-9-C25:1 was more abundant (see Fig. 4a). This polymorphic variation in the production of one or the other alkene in females of *P. triangulum* within the same population had already been reported before [28, 50]. It would be interesting to test the hypothesis that this chemical polymorphism is an adaptation to partly escape chemical mimicry by *H. rutilans*.

### CHC profile diversification is more pronounced in females than in males of COLw hosts

We hypothesized that females of COLw hosts responded stronger to selection pressure exerted by the brood parasites on their CHC profile and thus show a greater CHC profile diversity than males of COLw hosts. If COLw species are able to evolve CHC profiles that differ from that of their brood parasites, we should see this pattern more pronounced in females than in males, assuming that CHC profiles evolve sex-specifically. While CHC profiles of the analyzed hosts do not show striking sex-specific qualitative differences, irrespective of whether we analyzed COLw or HYMw, we found quantitative differences between the CHC profiles of females and of males. Specifically, females of COLw are characterized by possessing larger proportions of methyl-branched alkanes than their conspecific males on their cuticle. In the COLw species *C. arenaria,* females furthermore synthesize a larger number of methyl-branched compounds than conspecific males. Male-exclusive methyl-branched compounds that contribute to CHC diversity in male COLw of *C. interrupta* and *C. quinquefasciata* (Additional file 1: Fig. S1a,b) were not produced in large amounts and made up less than 3% of the total CHC profile in males. Overall, the CHC profile differences between males and females are consistent with the idea with female COLw hosts escaping chemical mimicry of their brood parasites.

### CHC profiles of female cuckoo wasps parasitizing COLw hosts are more similar to those of their hosts than to those of conspecific males

We hypothesized that only cuckoo wasp females have been selected to mimic the CHC profile of their female hosts. In contrast, cuckoo wasp males should not have been selected to mimic the CHC profile of their female hosts due to the lack of interaction with their host or their host’s nest. This pattern should be particularly recognizable in COLw and their brood parasites, because COLw are free from selection to synthesize alkene-enriched CHC profiles due to the abandonment of prey embalming behavior [37]. As predicted, we found that cuckoo wasp females parasitizing COLw species (with the notable exception of *H. chalybaeum*) largely synthesize the same type of compounds as their female COLw hosts (especially the same homologous series of methyl-branched alkanes). In contrast, male cuckoo wasps, irrespective of what type of host they develop from (*i.e.*, COLw or HYMw), show alkene rich CHC profiles that are compositionally similar to the CHC profiles of HYMw. Interestingly, a phylogenetic study of the host species gives evidence that HYMw species are ancestral to COLw [37]. Thus, males of all cuckoo wasps seem to still synthesize a more ancestral CHC profile and are evolutionarily less affected by the shift towards higher quantities of methyl-branched hydrocarbons in COLw than are cuckoo wasp females.

## Conclusions

Brood parasitism can exert strong selection pressure on the host to improve its ability to detect brood parasites. The brood parasites, in turn, are counter-selected for traits that enable them to remain undetected by their host. The switch from using Hymenoptera to using Coleoptera as prey in Philanthidae liberated COLw from producing CHC profiles enriched with unsaturated CHCs, a requirement for embalming and preserving Hymenoptera prey. We demonstrated that this relaxation likely allowed COLw species to evolve distinct and species-specific CHC profiles dominated by methyl-branched alkanes as a strategy to escape chemical mimicry by their *Hedychrum* brood parasites. Several lines of evidence are consistent with our conclusions: (1) COLw CHC profiles are conspicuously distinct from the alkene-enriched CHC profiles of HYMw necessary for prey preservation and very distinct between each species, with species-specific types of methyl-branched alkanes; (2) the CHC profile overlap between COLw and their brood parasites is smaller than between HYMw and their brood parasites, although the Bray–Curtis dissimilarities of the CHC profiles were not significantly smaller in HYMw compared to COLw; (3) female cuckoo wasps show a CHC profile that resembles that of their COLw host species (except for *H. chalybaeum*), whereas the CHC profile of their male conspecifics resembles the host’s likely ancestral CHC profile of HYMw. In addition, COLw species show CHC profiles with longer chain lengths than HYMw and all *Hedychrum* species, which may constitute an additional strategy to escape chemical mimicry by their brood parasites. In summary, the observed patterns suggest that a diversification of CHC profiles with methyl-branched alkanes has evolved in COLw host species and that their cuckoo wasps “followed” their hosts in chemical space, consistent with an arms race hypothesis. Furthermore, our data suggest that HYMw may counteract the chemical mimicry of cuckoo wasps by exhibiting a larger intraspecific CHC profile variability than COLw. Although we provide evidence for a coevolutionary arms race on the phenotypic level, future studies should consider searching for footprints of this arms race also at the genetic level.

## Methods

### Collection and origin of the insect samples

We studied the CHC profiles of 5–14 individual wasps per species and sex of twelve species (five species of the genus *Hedychrum*, five species of the genus *Cerceris*, and two species of the genus *Philanthus*, Table 2). To avoid considering individuals from populations that have experienced very different climatic conditions in the past, we collected all specimens in the Upper Rhein valley between Freiburg and Germersheim (150 km). These samples were collected over the course of multiple years, as some species are considered rare or difficult to find in larger numbers (*e.g*., *Hedychrum chalybaeum*). As sampling over multiple years could potentially increase variation (compared to sampling in just one year), we intentionally also collected the more common species over the course of multiple several years. We screened for possible differences in CHC profile composition between samples collected at different sample locations and in different years but did not find any significant differences (data not shown). Besides the five target species of *Hedychrum* (see above), we included an additional species of this genus (*i.e.*, *H. longicolle* collected in Almarail, Spain in 2011) in the phylogenetic analysis for reasons unrelated to the present study. All samples were collected with an insect net and were identified by ON using a reference collection and DNA barcoding. Each wasp was placed in a glass vial (1.5 mL) and transported to the lab, where the specimen was killed by freezing. All specimens were stored at − 20 °C until extracting their CHCs and (only in a subset of samples) their DNA. Note that CHCs were extracted prior to any molecular work. Voucher specimens of samples whose DNA we studied are deposited at the Zoological Research Museum Alexander Koenig in Bonn or in the personal collections of ON and TS (Additional file 2: Table S1). Species of *Hedychrum* are known to be very host specific, and the currently known host associations of the studied species is summarized in Table 3.

**Table 2:** Number of females and males, locality in Germany, and year of collection of specimens analyzed in this study

Species	Females	Males	Locality	Year of Collection
*Cerceris arenaria*	8	11	Kapsweyer	2009
*Cerceris interrupta*	8	6	Guntersblum	2011, 2012, 2014
*Cerceris quinquefasciata*	14	11	Kaiserstuhl	2005–2011
*Cerceris rybyensis*	14	14	Freiburg, Kaiserstuhl	2005, 2006, 2010, 2011
*Cerceris sabulosa*	14	14	Kaiserstuhl	2005, 2006, 2010, 2011
*Philanthus triangulum*	9	12	Kaiserstuhl	2005–2011
*Philanthus coronatus*	5	14	Kaiserstuhl	2005–2011
*Hedychrum chalybaeum*	9	10	Guntersblum	2011
*Hedychrum gerstaeckeri*	14	14	Kaiserstuhl	2005–2011
*Hedychrum nobile*	13	14	Kapsweyer	2009
*Hedychrum niemelai*	11	10	Albersweiler, Alsheim, Battensberg, Eisenberg, Guntersblum, Landau i. d. Pfalz (Ebenberg), Osthofen, Schwegenheim	2011–2014
*Hedychrum rutilans*	14	14	Kaiserstuhl	2005-–2011

**Table 3:** Species of *Hedychrum* and their hosts, as cited in taxonomic descriptions/reviews

	Apidae					Crabronidae												Vespidae	
	*Megachile parietina*^*1*^	*Osmia nigriventris*	*Lassioglossum leucozonium*	*Halictus quadricinctus*	*Halictus scabiosae*		*Bembicinus tridens*	*Oxybelus haemorrhoidalis*	*Dinetus pictus*	*Cerceris arenaria*	*Cerceris interrupta*	*Cerceris quadrofasciata*	*Cerceris quinquefasciata*	*Cerceris ruficornis*	*Cerceris rybyensis*	*Cerceris sabuosa*	*Philanthus coronatus*	*Philanthus triangulum*	*Ancistrocerus parietum*
Hedychrum chalybaeum							[76, 78, 82]		[82]		[67, 84–86]								
Hedychrum gerstaeckeri				[76]				[76, 78]						[86]	[75, 76, 78, 82, 83, 85, 86]	[67, 76], [85]	[67, 76] [78, 81]	[67, 76, 78, 81]	
Hedychrum niemelai										[67, 79, 86]		[86]	[67, 79, 83, 86]	[67, 79, 86]	[67, 78, 79, 86]				
Hedychrum nobile	[76, 78]	[76]	[76]							[67, 75, 76, 78, 82, 83, 85, 86]		[86]	[82]	[67]	[86]				[76]
Hedychrum rutilans					[76]												[76, 78, 79, 81, 85]	[67, 76–81, 83, 85, 86]	

The numbers refer to the references that cite host-parasite relationships. Confirmed or very likely host-parasite relationships marked in grey. Note that species name in some publication is different: ^1^*Chalicodoma murina*, ^2^*Halictus leucozonius*, ^3^*Halictus quadristrigata*, ^4^*Halictus zebrius*, ^5^*Stizus tridens*, ^6^*Oxybelus elegantulus*, ^7^*Cerceris emaginata*, ^8^*Odynerus parietum*

### Chemical analysis

Frozen insects were allowed to thaw for about five minutes and were subsequently submerged in pure n-hexane (Merck, Darmstadt, Germany) to extract the CHCs. After 10 min, the CHC extract was transferred into another glass vial, concentrated with a gentle stream of CO_2_ until approximately 80–100 µL of the solvent remained. The CHC extract was stored at − 20 °C, and the insect was stored separately in 100% ethanol to preserve its DNA.

A HP 6890 gas chromatograph (GC) coupled with a HP 5973 Mass Selective Detector (MS) (Hewlett Packard, Waldbronn, Germany) and an Agilent 7890/5975 GC/MS System were used for analyzing the extracts. The GCs (split/splitless injector in splitless mode for 1 min, injected volume: 1 µl at 300 °C injector temperature) were equipped with a DB-5 Fused Silica capillary column (30 m × 0.25 mm ID, df = 0.25 µm^2^, J&W Scientific, Folsom, USA). Helium was used as carrier gas with a constant flow of 1 mL/min. We applied the following temperature program: start temperature at 60 °C, with an increase of 5 °C/min until 300 °C, and isotherm at 300 °C for 10 min. An ionization voltage of 70 eV (source temperature: 230 °C) was set for the acquisition of the mass spectra by electron ionization. Ion masses of 40–600 units were recorded.

In order to identify the double-bond position of alkenes, 1–5 extracts of each sex and species (depending on the available amount of CHC extract) were pooled and used for dimethyl disulfide (DMDS) derivatization following the protocol provided by Carlson and colleagues [51]. The double bond positions of alkadienes remained unidentified due to the low quantity of this substance class in the CHC extracts. Alkadienes were characterized according to their retention indices.

We analyzed the cuticular hydrocarbon composition of cuckoo wasps and their hosts with the software AMDIS version 2.71 (Automated Mass Spectral Deconvolution and Identification System, http://chemdata.nist.gov/mass-spc/amdis/). AMDIS uses mass spectra similarities and retention indices to select target compounds. Specifically, we first created a mass spectral library (which contains more than 600 identified mass spectra of common hydrocarbons and their retention indices). We used the protocol by Carlson and colleagues [52], in which the elution patterns of CHC are described to confirm the identified methyl-branched alkanes in this library. The parameters used in AMDIS were as follows: component width = 22, adjacent peak subtraction = 2, resolution = medium, sensitivity = low, and shape requirements = medium.

After CHC had been identified, we calculated their relative abundance by dividing the total ion count of each peak relative to the total ions count of CHC in a profile occurring in the range of C21–C33. To ensure that a compound occurred in the majority of samples of a given species and did not represent an artifact of CHC extract concentration differences or of the sensitivity of the GC/MS, we set a threshold for the consideration of any compound within a group. This minimum threshold requirement was met if the compound occurred in at least 50% of all specimens (per sex and species) and if the mean relative quantitative abundance was at least 0.1%.

We estimated the total number of compounds and the number of compounds per compound class (*i.e*., linear alkanes, unsaturated compounds, and methyl-branched alkanes). Additionally, we calculated the mean chain length of the CHCs in a given CHC extract by summing up the relative amount of each peak within the range C21 to C33, weighted by its retention index. This value indicates the retention index at which half of the relative amount of the CHC profile occurs.

### Statistical analyses of chemical data

CHC profiles were compared and differences were visualized using multivariate methods using the software R version 3.02 [53]. We conducted Non-metric Multidimensional Scaling (NMDS) using Bray–Curtis dissimilarity to visualize CHC profile similarity in two-dimensional graphs [54, 55]. All inferred stress values fell below 0.15. We assessed the degree of similarity and overlap between CHC profiles of the different groups using an analysis of similarity (ANOSIM; [56], a non-parametrical test that operates on a ranked dissimilarity matrix. The obtained test statistic R (− 1 < R < 1) indicates the degree of similarity between and within groups. An R value close to 1 indicates complete separation between the tested groups and values close to zero indicates more similarity between the groups (greater overlap, less separation). Negative values of R are uncommon and do not have a biological meaning.

We used Welch corrected t-tests [57, 58] to compare various traits between COLw and HYMw and their brood parasites. Specifically, we compared Bray–Curtis dissimilarities between hosts and their brood parasites, the proportion, number, and diversity of CHC compounds, and the mean chain length. P values from the ANOSIM tests and the t-tests were adjusted using the Holm-Bonferroni correction [59] to account for multiple testing.

For most of the analyses, we used the R package *vegan* [60]. R packages used for plotting were *ade4* [61], *ape* [62], *flagme* [63], *phytools* [64], and *xcms* [65].

### Molecular procedures

We used muscle tissue from one or two individuals per species for DNA extraction (see below) after CHC extraction.

We used a set of degenerated oligonucleotide primer pairs [66] to amplify twelve single-copy protein-coding nuclear genes in cuckoo wasps (same as studied by [67]; Additional file 2: Table S2). Polymerase chain reactions (PCR) and bidirectional direct Sanger sequencing followed the protocol given by [67].

Forward and reverse DNA strands were assembled to contigs and trimmed (to exclude binding sites of PCR primers) in Geneious (version 6.1; [68]. All contigs were aligned with the LINS-i algorithm of MAFFT version 7.123 [69]. Intronic and exonic regions were annotated manually by aligning a reference DNA sequence of each target gene from the 1KITE transcriptome of the cuckoo wasp *Chrysis terminata* and identifying canonical splice sites in the genomic DNA sequencing (*i.e.*, the dinucleotide pair GC-AG) (see [67]. We manually removed uninformative and ambiguously aligned sites from the intronic DNA sequences. Finally, we concatenated all exons and introns to a supermatrix and defined three partitions: (1) 1st and 2nd codon position of exons, (2) 3rd codon position of exons, (3) introns.

### Phylogenetic analyses

We inferred the phylogenetic relationships of the studied *Hedychrum* species by applying the maximum likelihood optimality criterion. Specifically, we first searched for the best fitting substitution model for each partition using Modelfinder [70] implemented in IQ-TREE (version 1.5.5; [71]) and applying the corrected Akaike information criterion (AICc) to choose between models. The partition-specific substitution models are listed in Additional file 2: Table S3. The selected substitution models and the empirically inferred substitution parameters were subsequently used to infer a phylogenetic tree within IQ-TREE. Statistical branch support was estimated from 1000 non-parametric bootstrap replicates. We additionally conducted a phylogenetic analysis in a Bayesian framework with the software MrBayes version 3.1.2 [72, 73]. For this purpose, we repeated the model selection step in Modelfinder, but restricting the tested models to those implemented in MrBayes. We started two parallel runs, both with a random starting tree, over 10^7^ generations. We sampled trees every 10^5^ generation and discarded the first 10^6^ generations of both runs as burn-in. We used the remaining trees from all runs in order to calculate a 50% majority rule consensus tree. Convergence was assessed with the software Tracer (version 1.6; [74]).

To infer the phylogenetic relationship of *Cerceris* and *Philanthus* species, we use the phylogeny published by Wurdack and colleagues [37] and trimmed it to the species analyzed in the present study.

## Supplementary Information

**Additional file 1: Figure S1.** Boxplots of the number of a) total CHC compounds and b) methyl-branched alkanes and the c) proportion of methyl-branched alkanes in the profiles of Hymenoptera preying (HYMw) and Coleoptera preying (COLw) apoid wasps, hosts to *Hedychrum* species in this study. Females are depicted in dark grey and males in light gray colors. **Figure S2.** Intra- and interspecific variability of cuticular hydrocarbon profiles in a) female and b) male individuals of all host species. Bray–Curtis dissimilarities were calculated between all individuals of species hunting the same type of prey (“between species”) and between individuals of the same species (species name indicated). In this case, however, in comparison to the figure presented in the text, only a maximum of five individuals were randomly selected for each group so that the number of specimens used in each group remains the same (5). **Figure S3.** Bray–Curtis dissimilarities between two randomly selected CHC profiles of samples collected from the same population of a species belonging to HYMw or to COLw in females (a) and males (b). Pairwise distances between two individuals coming from the same species were randomly selected in 1000 simulations in each case.

**Additional file 2: Table S1.** Provenance and details of collection and storage of all specimens used in the analyses. **Table S2.** Characteristics of the primers used and number of nucleotides in the exonic regions of the nuclear genes used for the phylogenetic analyses. Numbers before the semicolon indicate number of exonic regions, and the numbers following the semicolon indicate the total number of nucleotides used per gene. **Table S3.** Substitution models chosen by Modelfinder for the respective phylogenetic analysis in IQTree and MrBayes. **Table S4.** Mean relative abundance ± standard deviation for each peak or mixed peak included in the NMDS of the females of all species analyzed. **Table S5.** Mean relative abundance ± standard deviation for each peak or mixed peak included in the NMDS of the males of all species analyzed. **Table S6.** Accession numbers for the genes used for the molecular phylogeny in Fig. 1. **Table S7.** Relative composition of the CHC compounds of all specimens. First row shows retention index with compound name abbreviation. First column shows the specimens name, sex, and number

## Data Availability

Voucher specimens are deposited in the repositories listed in Additional file 2: Table S1. Accession numbers for the genes used for the molecular phylogenies are listed in Additional file 2: Table S6. Relative compositions of the CHC compounds of all specimens are listed in Additional file 2: Table S7.

## Abbreviations

CHC: Cuticular hydrocarbon
COLw: Coleoptera-hunting wasps
HYMw: Hymenoptera-hunting wasps
GC/MS: Gas chromatography/mass spectrometry
