# Supplementary material for: Evidence for a chemical arms race between cuckoo wasps of the genus Hedychrum and their distantly related host apoid wasps
Source: BMC Ecol Evol. 2022 Nov 28;22:138. doi: 10.1186/s12862-022-02093-8 (PMC9703671; doi:10.1186/s12862-022-02093-8)
Supplement: Supplementary file 1 — Additional file 1: Figure S1. Boxplots of the number of a) total CHC compounds and b) methyl-branched alkanes and the c) proportion of methyl-branched alkanes in the profiles of Hymenoptera preying (HYMw) and Coleoptera preying (COLw) apoid wasps, hosts to Hedychrum species in this study. Females are depicted in dark grey and males in light gray colors. Figure S2. Intra- and interspecific variability of cuticular hydrocarbon profiles in a) female and b) male individuals of all host species. Bray–Curtis dissimilarities were calculated between all individuals of species hunting the same type of prey (“between species”) and between individuals of the same species (species name indicated). In this case, however, in comparison to the figure presented in the text, only a maximum of five individuals were randomly selected for each group so that the number of specimens used in each group remains the same (5). Figure S3. Bray–Curtis dissimilarities between two randomly selected CHC profiles of samples collected from the same population of a species belonging to HYMw or to COLw in females (a) and males (b). Pairwise distances between two individuals coming from the same species were randomly selected in 1000 simulations in each case. [file 12862_2022_2093_MOESM1_ESM.docx]

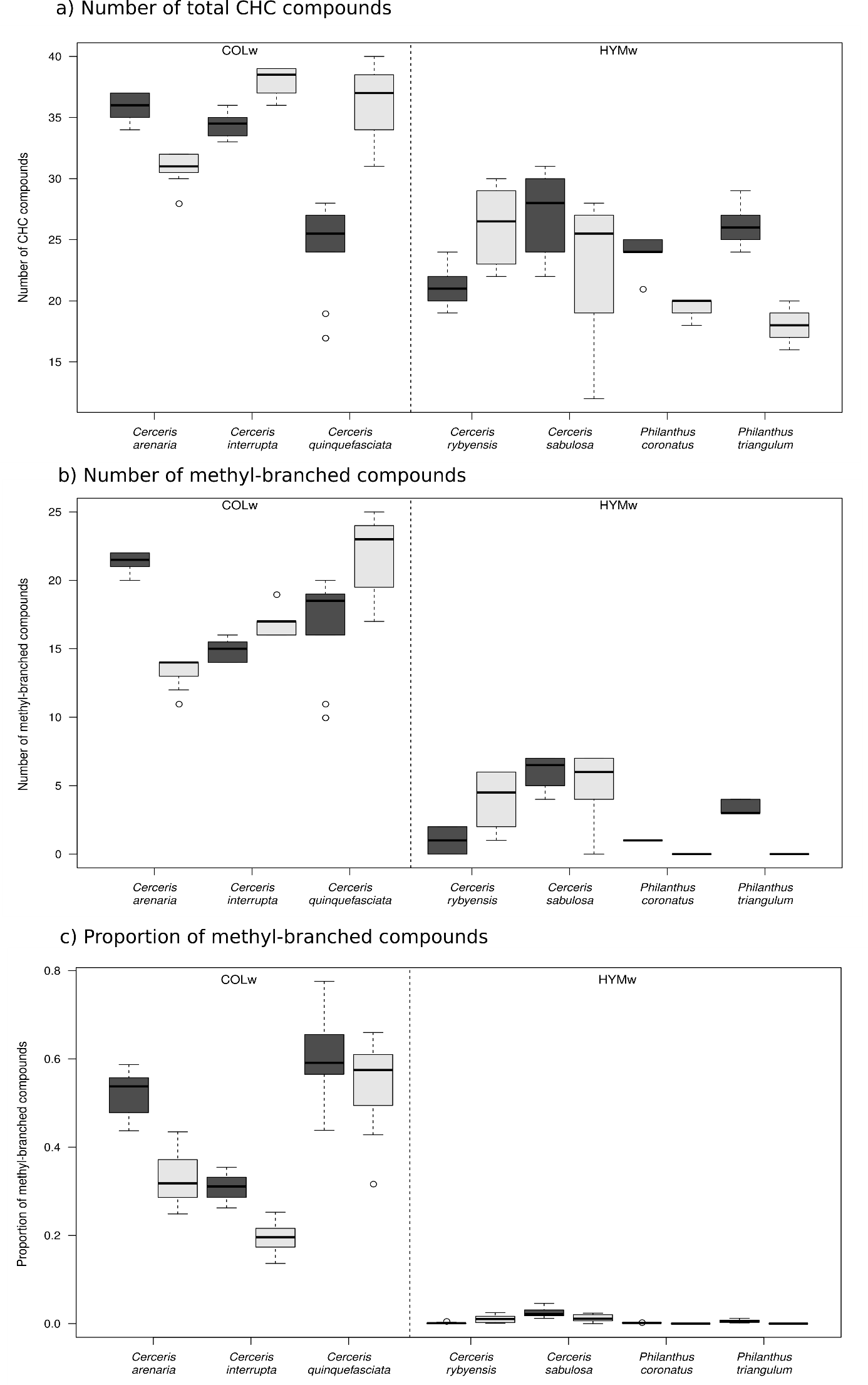


SM-Figure 1: Boxplots of the number of a) total CHC compounds and b) methyl-branched alkanes and the c) proportion of methyl-branched alkanes in the profiles of Hymenoptera preying (HYMw) and Coleoptera preying (COLw) apoid wasps, hosts to *Hedychrum* species in this study. Females are depicted in dark grey and males in light gray colors.


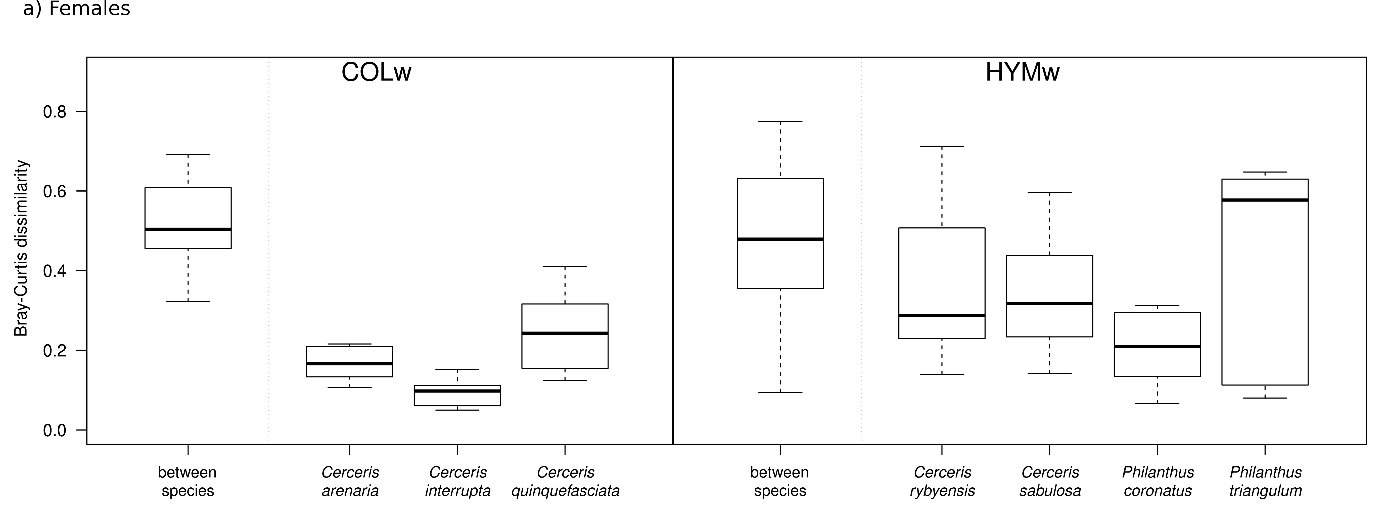


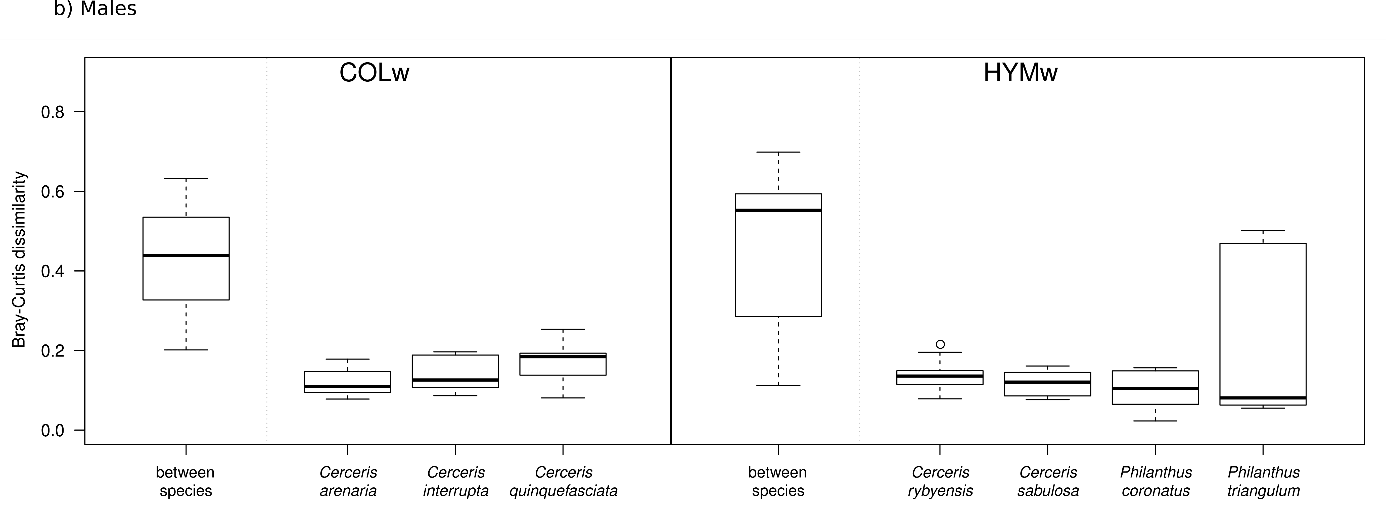


SM-Figure 2: Intra- and interspecific variability of cuticular hydrocarbon profiles in a) female and b) male individuals of all host species. Bray-Curtis dissimilarities were calculated between all individuals of species hunting the same type of prey (“between species”) and between individuals of the same species (species name indicated). In this case, however, in comparison to the figure presented in the text, only a maximum of five individuals were randomly selected for each group so that the number of specimens used in each group remains the same (5).


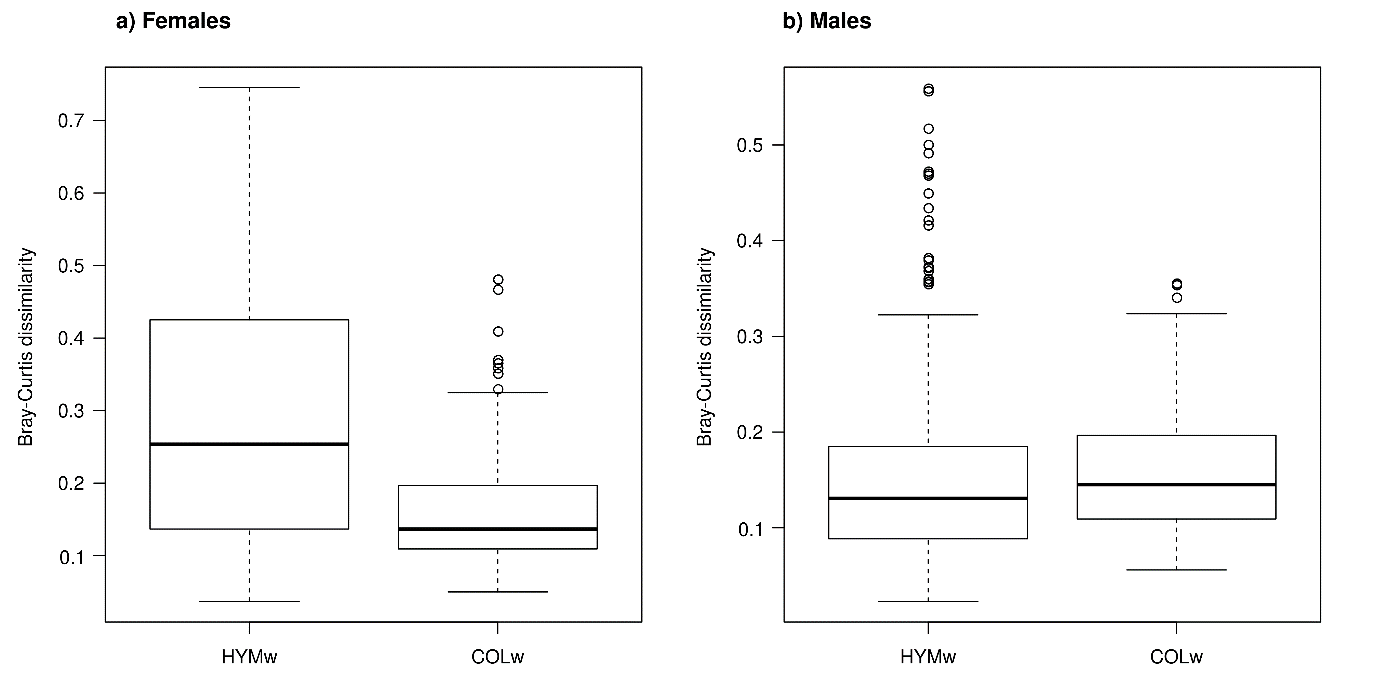


SM-Figure 3: Bray-Curtis dissimilarities between two randomly selected CHC profiles of samples collected from the same population of a species belonging to HYMw or to COLw in females (a) and males (b). Pairwise distances between two individuals coming from the same species were randomly selected in 1000 simulations in each case.
